# Bibliographical

**Published:** 1867-11

**Authors:** 


### BIBLIOGRAPHICAL.

*Catalogue of the United States Army Medical Museum*—Prepared under the direction of the Surgeon General, U. S. Army.

The above quarto volume of 960 pages was sent us from the Surgeon General's office. It is arrganged regularly in Surgical, Medical and Microscopical Sections.

This work, as the title implies, is merely a systematically arranged catalogue of the Army Medical Museum, giving the character and number of specimens in each section of the Medical Museum at Washington.

The chief features of the book consist in the discription, and, in some instances, plates of interesting pathological and microscopical specimens. It is of no very great practical importance to physicians, but will please the curious.

*Circular No. 7.—A Report on Amputations at the Hip-joint, in Military Surgery.* By Geo. A. Otis, Assistant Surgeon and Brevet Lieut. Colonel, U. S. Army.

This is a stitistical account of all the cases in which this operation was performed, in the Confederate and United States Armies, that have been reported to the War Department at Washington, and will be found interesting to the reader.

*Studies in Pathology and Therapeutics.* By SAMUEL DICKSON, M. D., L. L. D., Professor of Practice of Physic, in Jefferson Medical College, &c., &c.

This little volume of 200 pages, from the Publishing House of Wm. Wood & Co., New York, was furnished us by the publishers.

The author, in his remarks on disease—its character and tendency—says that which should be read and understood by every practitioner of medicine. Without correct views on this subject we cannot prescribe rationally, and yet its study is lamentably neglected by the busy practitioner.

The twenty-five pages devoted to therapeutics are of themselves worth the price of the book. Prof. Dickson is not swerved from a rational course in the treatment of disease, by hypothetical innovations on practical and substantial truths.

*On the Action of Medicines in the System.* By FREDERICK WILLIAM HEADLAND, M. D., B. A., F. L. S., Fellow of the Royal College of Physicians, &c.

The above work of 430 pages was forwarded us by the publishers, Lindsay & Blakiston, Philidelphia.

While we differ with the author on many points connected with the action of remedies, we must accord to him a degree of thoroughness of investigation, to which few writers have attained. We have been profited and pleased in its perusal. Its popularity may be inferred from its having reached the fifth American, from the fourth London edition—revised and enlarged.

*Chemistry.* By WILLIAM THOMAS BRANDE, D. C. L., &c. &c., and ALFRED SWAINE TAYLOR, M. D., &c. &c.—Second American Edition; thouughly revised. HENRY C. LEA, publisher—Philadelphia, 1867.

This work doubtless comes up to the times, and as advancement in the science of chemistry is constantly being made, it becomes the interest of the student to consult recently revised works.

